# Development of Public Health Core Outcome Sets for Systems-Wide Promotion of Early Life Health and Wellbeing

**DOI:** 10.3390/ijerph19137947

**Published:** 2022-06-28

**Authors:** Liina Mansukoski, Alexandra Albert, Yassaman Vafai, Chris Cartwright, Aamnah Rahman, Jessica Sheringham, Bridget Lockyer, Tiffany C. Yang, Philip Garnett, Maria Bryant

**Affiliations:** 1 Department of Health Sciences, University of York, York YO10 5DD, UK; yassaman.vafai@york.ac.uk; 2 Thomas Coram Research Unit, University College London (UCL), London WC1H 0AL, UK; a.albert@ucl.ac.uk; 3 Bradford Institute for Health Research, Bradford Teaching Hospitals NHS Foundation Trust, Bradford BD9 6RJ, UK; chris.cartwright@bthft.nhs.uk (C.C.); aamnah.rahman@bthft.nhs.uk (A.R.); bridget.lockyer@bthft.nhs.uk (B.L.); tiffany.yang@bthft.nhs.uk (T.C.Y.); 4 Department of Applied Health Research, University College London (UCL), London WC1E 6BT, UK; j.sheringham@ucl.ac.uk; 5 School of Management, University of York, York YO10 5DD, UK; philip.garnett@york.ac.uk; 6 Hull York Medical School, University of York, York YO10 5DD, UK

**Keywords:** early life health, core outcome set, public health interventions, systems approach

## Abstract

We aimed to develop a core outcome set (COS) for systems-wide public health interventions seeking to promote early life health and wellbeing. Research was embedded within the existing systems-based intervention research programme ‘ActEarly’, located in two different areas with high rates of child poverty, Bradford (West Yorkshire) and the Borough of Tower Hamlets (London). 168 potential outcomes were derived from five local government outcome frameworks, a community-led survey and an ActEarly consortium workshop. Two rounds of a Delphi study (Round 1: 37 participants; Round 2: 56 participants) reduced the number of outcomes to 64. 199 members of the community then took part in consultations across ActEarly sites, resulting in a final COS for systems-based public health interventions of 40 outcomes. These were grouped into the domains of: Development & education (N = 6); Physical health & health behaviors (N = 6); Mental health (N = 5); Social environment (N = 4); Physical environment (N = 7); and Poverty & inequality (N = 7). This process has led to a COS with outcomes prioritized from the perspectives of local communities. It provides the means to increase standardization and guide the selection of outcome measures for systems-based evaluation of public health programmes and supports evaluation of individual interventions within system change approaches.

## 1. Introduction

### 1.1. Background and Objectives

Core outcome sets (COS) are “an agreed standardized collection of outcomes” used in evaluations of intervention research [1]. The use of COS has been promoted to harmonize the outcomes used and to ensure that key stakeholders are consulted on the relevance of what is being measured in evaluations [2]. No existing core outcome set has been adapted specifically for the systems-wide promotion of early life health and wellbeing in public health research in the UK, two widely used outcomes frameworks are the Public Health Outcomes Framework (PHOF) and the NHS Outcomes Framework [3,4]. Though an important resource to highlight key indicators to measure the success of some early life interventions, the most widely used existing framework for public health, the PHOF, was not developed to ensure the use of a minimum set of outcomes to be used across studies to facilitate comparisons. Most COS in the pediatric literature, on the other hand, focus on a specific illness or disease, not on public health outcomes [5].

We sought a COS to support the evaluation of a UKPRP-funded programme of research called ActEarly. ActEarly is a large research consortium aimed at promoting health and wellbeing in early life in two different areas with high rates of child poverty: Bradford in West Yorkshire and the Borough of Tower Hamlets in London [6]. Living in an area with high levels of child poverty often coincides with exposure to other economic, physical, cultural, learning, social and service environmental risk factors, which can predispose children and their families to poorer mental and physical health outcomes. In 2019, ActEarly was launched to address these issues with the aim of creating testbeds of upstream interventions within ‘whole system city settings’ (i.e., understanding and addressing the interconnectedness of distal and proximal determinants) [6,7]. The programme is a partnership between academics, local governments, the NHS, Bradford Institute for Health Research, community and third sector organizations and staff/students at affiliated universities (University of York, Leeds, Bradford, Queen Mary University London, University College London, London School of Hygiene and Tropical Medicine). The ActEarly programme combines interventions with citizen science and the co-production of research with local communities across the two study sites [8,9].

As ActEarly is a system-wide intervention, it necessitates system-wide outcome sets that incorporate multiple aspects of health, well-being and the physical and social environment in which the families and children of Bradford and Tower Hamlets live. The COS was deemed essential, not only to ensure consistency and comparability in what is being measured by planned project evaluations within ActEarly, but to facilitate a system-wide meta-evaluation of the whole ActEarly programme, including planned long-term economic modelling [10]. The lack of an agreed set of core public health outcomes specific to early years and childhood health and well-being that takes a whole-systems perspective was identified as a key gap in our evaluation work in this area. Rather than providing a wider selection of outcomes (i.e., similar to the PHOF framework), the COS presented here was intended to represent the ‘minimum’ required set of outcomes (though not necessarily excluding the inclusion of other outcomes). Thus, we aimed to develop the public health ‘Core Outcome Set for Early Years (COS-EY)’. The specific objectives of this COS development were to:

1. Identify an agreed minimal dataset of potential outcomes from locally relevant frameworks.
2. Achieve expert consensus on the COS through a two-stage Delphi consultation process.
3. Incorporate the perspective of the local communities in which early years and childhood interventions are targeted, in the COS development.
4. Arrive at a final COS-EY.

### 1.2. Scope

To define the scope of the COS development, we followed the Core Outcome Measures in Effectiveness Trials (COMET) guidance [2]. However, rather than targeting a specific health condition, we extended our scope to include outcomes that would be deemed important across the whole system. Given the intended breadth of this work, we therefore anticipated that we would develop a series of combined COS within domains such as: Social environment, Physical health, Poverty, etc. Thus, although our goal was to develop an overarching systems-based COS, we also anticipated developing domains, and that each of the domains would generate a separate sub-COS consisting of a smaller set of outcomes (~three to seven).

### 1.3. Interventions

The development of the COS-EY was guided by ActEarly’s three themes (Healthy places; Healthy livelihoods; and Healthy learning) and four cross-cutting themes (Food & healthy weight; Play and physical activity; Co-production and Citizen science; and Evaluation). Each theme consists of multiple projects located across the two study sites. Examples of ActEarly projects include an evaluation of the Healthy School Streets programme in both Bradford and Tower Hamlets; the Join Us: Move. Play (JU:MP) local delivery pilot which aims to test and learn more about what helps children aged 5–14 years to be active; and co-production of the Horton Park regeneration project in Bradford (for further details of these and other ActEarly projects, see [11]). There are no constraints placed on potential study designs and there is a great variety of approaches taken within ActEarly to achieve the overall goal of early promotion of good health and wellbeing. This means the process to develop the COS needed to be flexible and fit for purpose to accommodate different study designs, populations and evaluations.

## 2. Materials and Methods

Guided by the principles set out in the COMET (Core Outcome Measures in Effectiveness Trials) Handbook [2], we designed a modified Delphi study consisting of two rounds of a consensus survey administered to our panel of experts and stakeholders, followed by a face-to-face public consultation with community members using ‘dot voting’ (details below). The Delphi method was first developed by the RAND corporation and is commonly used to create consensus by asking participants to answer questions across multiple rounds. After each round, responses are fed back to the participants [2,12]. The decision to start the process with the expert and stakeholder consultation, followed by the community consultation, was taken because of their knowledge of interventions and the whole system changes needed to be seen.

### 2.1. Registration

The COS development was registered on the COMET website (#1910) and the reporting of the study is in line with the COS-STAR Statement [13,14].

### 2.2. Participants

**Figure 1:**
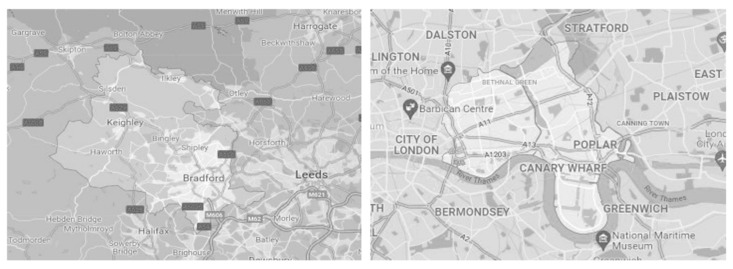
Maps of ActEarly study areas: Bradford Metropolitan District (**left**) and the London Borough of Tower Hamlets (**right**).

The populations that are the targets for the application of the COS-EY in the first instance were children and families living within the ActEarly study areas: Bradford Metropolitan Area in West Yorkshire and the Borough of Tower Hamlets in London (Figure 1).

Stakeholder groups who were involved in the COS development included: ActEarly researchers, community and council partners and community members in Bradford and Tower Hamlets. This wide consultation allowed us to consider the viewpoints and expertise of academics, as well as affiliated local government and public health professionals. In addition, it was considered vital that the communities in which ActEarly operates were consulted to prioritize the evaluation of changes in factors that were important and meaningful to the families and children living in each local area.

For the first round of the survey, anyone within the immediate or wider ActEarly team, including academics, practitioners, local government, voluntary sector organizations and community representation, was eligible to take part (due to the snowball sampling, it is not possible to provide a precise sample size of how many people were invited to take part in the Delphi surveys but we estimate that the link to the survey may have reached anywhere between 70 to 100 people).

For round two, the eligibility criteria stayed the same, but we extended our promotion and reach in an attempt to get wider participation. At this point, the project had grown in size and reach and we felt it was important to ensure individuals who had newly joined, or newly become collaborators, had the opportunity to contribute to the COS development. Potential participants were identified from the activity logs of the ActEarly projects and by asking ActEarly theme-leads to signpost key collaborators and partners, local government links and members of the communities associated with ActEarly and other related projects.

The eligibility criteria for participation in the community consultations were purposefully left open and included any adult attending any of the events at which the consultations took place. To widen the reach of the consultation, we conducted all three consultations in open, public areas. In Bradford, this included Horton Park and Peel Park. Both parks held free entry events that were visited by local children and families over the summer of 2021. In Horton Park, the event was an Eid celebration aimed at local families. In Peel Park, the event was a council-funded Play Bradford event. We estimate that each event was attended by 100+ local families but do not have exact figures. We did not collect demographic, social or health information from the families but most participants arrived at the events on foot from the surrounding neighborhoods. In Tower Hamlets, the consultation was conducted in collaboration with the Bromley by Bow Centre who identified the Old Ford Road Summer Fun Day event at Butley Court as suitable for the consultation.

### 2.3. Information Sources (Development of the Minimal Dataset)

**Figure 2:**
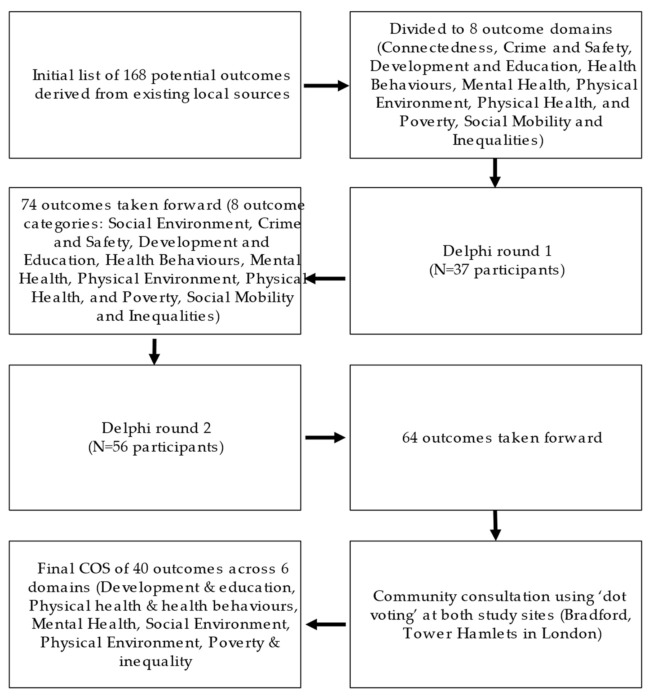
Process to reduce the number of outcomes.

The initial list of potential outcomes was derived from existing local sources including: the Bradford Key Indicators set; Tower Hamlets key indicators; Tower Hamlets ‘I’ statements (publicly derived framework); the Tower Hamlet common outcomes framework; ActEarly community survey codes; and individual suggestions from stakeholders at previous ActEarly workshops (Figure 2). This process involved collating all outcomes from each of these local sources, in which the words and presentation of text were retained. Outcomes which were repeated by more than one source (e.g., childhood obesity) were only included once in the minimal dataset. However, those deemed to be ‘similar’, but not identical, were retained as separate outcomes (e.g., ‘mental health’ and ‘mental well-being’). The listed outcome sources were developed locally and are regularly updated (thus links cannot be provided).

### 2.4. Consensus Process

#### 2.4.1. Surveys

The outcomes in the surveys were based on a collation of everything gathered from the activities in the ‘information sources (development of the minimal dataset)’ paragraph. Potential participants in the consensus surveys learnt about the study via email or word of mouth and snowballing of these (e.g., via existing groups/teams). The purpose of the study was summarized to participants on the first page of the survey to give context. The survey was completed using the online survey platform Qualtrics [15]. Invited participants received reminder emails. This survey asked participants to rate the importance of each outcome on a scale of 1–9 (from 1 “Not important at all” to 9 “Very important”). After all outcomes were rated, participants were asked to suggest any new outcomes not yet included. Our Delphi process did not include the collection of identifying information, but survey respondents were asked to state their stakeholder role (i.e., Academic, Clinical academic, Local government, Voluntary sector, Community representative, National/regional government, Commercial sector, Other).

The shortened Round 2 survey was also sent using Qualtrics. As in Round 1, invited participants received email reminders about the survey. In addition to asking participants to rate the importance of each survey, the Round 2 survey presented the group-average results of the first survey and encouraged participants to review these results before re-rating the outcomes. At the end of the Round 2 survey, there was an option to request outcomes that had been excluded after Round 1 to be re-introduced, as well as space to leave any other comments or suggestions.

#### 2.4.2. Community Consultation

**Figure 3:**
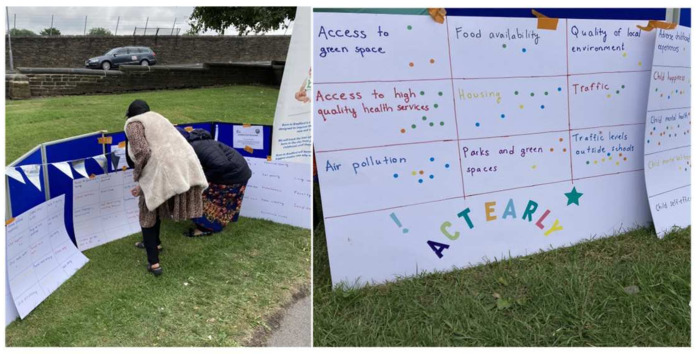
Community consultation in Bradford.

The final part of the consensus process was undertaken after the second survey had been analyzed (and the number of outcomes was hence reduced) with community members, that is, local families with children (Figure 3). In consensus methods, consultation with patients, or community members, is recommended when there is no clear consensus among the experts and it can ensure that outcomes are included that are important to community members [2]. The community member consultation was conducted using ‘dot voting’ and by utilizing principles of the nominal group technique, which facilitates quick, structured decision making [16,17,18]. In dot voting, participants are given colored dot stickers that they can use to indicate their votes in priority setting and consensus exercises. In addition to the ‘dot voting,’ we facilitated a play activity that children could engage in, whilst adults were asked to contribute to the core outcome consultation.

To make the process of voting as easy as possible, participants were asked to select and rank three outcomes they considered to be most important by placing their colored stickers on posters that included all the outcome names (green sticker for most important, yellow for second most important and orange for third most important outcome). The consultation facilitators (researchers) were present to answer any questions that arose and help explain the project and the outcomes that were voted on.

### 2.5. Analysis

#### 2.5.1. Outcome Scoring/Feedback

Survey items were scored on a 9-point Likert scale (where 1 was “Not important at all” and 9 was “Very important”). Although no definitive recommendation exists on the optimal number of points for a Likert scale in COS development, a 9-point Likert scale has been proposed for use in consensus processes to reduce the number of outcomes, before face-to-face consultations are taken to reach a final consensus [19]. The scores generated from Round 1 and Round 2 of the consensus surveys were analyzed using descriptive statistics (mean and median score, standard deviation, range) and by calculating expert agreement to identify which outcomes participants agreed were less important, outcomes for which there was good agreement for prioritizing and outcomes about which participants were uncertain.

The proportion of experts/stakeholders (details of participants in Table 1) agreeing was calculated as:

$$Proportion\;in\;agreement=\frac{N\;of\;experts\;scoring\;an\;item\;within\;a\;specified\;range}{Total\;N\;of\;experts}$$

The ‘proportion in agreement’ is sometimes referred to as the agreement index and multiplying the index by 100 results in the % of experts who agree with a given outcome based on our criteria set above.

All statistical analyses were performed using Stata 16 [20].

#### 2.5.2. Consensus Definition

To define consensus, we used ‘proportion within a range’. This definition of agreement is widely used in Delphi studies [21]. Agreement was defined as more than 80% of the panel scoring an item within a specified range on the 9-point Likert scale. Commonly, items scored as 1–3 are considered to indicate the outcome is of limited importance, items scored 4–6 are considered to be important but not critical and items scored 7–9 are deemed to be critical [2].

As recommended by the literature, we selected our agreement threshold of 80% in advance [22,23]. 80% is above the median threshold reported in the literature for the determination of consensus, which is 75% [21]. This slightly stricter threshold was selected due to the relatively large initial number of items in the Round 1 survey (N = 168), which needed to be reduced considerably to arrive at a feasible number of core outcomes. Disagreement was defined as <80% of the panel scoring an item within the specified range.

Thus, our process for keeping or removing outcomes applied the following rules:

1. Automatic inclusion: More than 80% of the participants scored the outcome 7, 8 or 9.
2. Automatic exclusion: More than 80% of the participants scored the outcome 1, 2, or 3.
3. For all remaining outcomes: the decision whether to include or exclude items from the subsequent round (Round 2) of the survey was considered following discussion within the immediate study team (M.B. & L.M.). Key considerations were the distance from the 80% automatic inclusion agreement index cut-off (% of experts ranking outcome 7 or higher); Round 1 median score; the balance of representation of outcomes across the outcome domains; and feedback from the open-ended comments made by participants in the survey. Adaptations to the approach were considered as appropriate based on the outcomes identified for the minimum dataset and how they were constructed, in addition to our need to reduce outcome lists to represent ‘core sets’ where participants were unable to deprioritize their importance.

This procedure was repeated with the Round 2 data following Round 2 survey implementation; however, we applied a less stringent inclusion cut-off (>70% of experts scoring 7 or higher) at this stage to provide members of the public in both communities with a large range of potential outcomes to consider. Missing observations (where an expert did not score a given outcome) were excluded from analyses.

#### 2.5.3. Community Consultation—Analysis

Following the dot voting process, outcomes were ranked by the number of votes by each study site with the aim of creating a ‘top 10′ ranking for each site. Each dot was given a score of 1 (dot color was not considered), and these were summed for each outcome. Outcomes ranked in the top 10 for each site were included in the final COS, even if the expert consensus on the given outcome was below the 80% cut-off (>80% of experts scoring the item 7 or higher) to signify the importance of public opinion.

### 2.6. Ethics

The University of York Department of Health Sciences Research Governance Board approved the study (reference: HSRGC/2021/458/E). Survey participants were asked to consent to take part. Community consultation did not collect any personal or identifiable information about the participants beyond the dot votes, and no informed consent was obtained.

## 3. Results

### 3.1. Participants

37 participants completed the Delphi questionnaire in Round 1 and 56 in Round 2. Due to us using snowball sampling when sending out the survey, we could not estimate how many of the people receiving the survey chose to participate in it. Participant stakeholder representation for the Delphi surveys is provided in Table 1, indicating that most respondents were academics or representatives from local government. A total of 199 members of the community took part in consultations (135 in total for the two events held in Bradford and 64 in total for the one event held in Tower Hamlets, London).

### 3.2. Outcomes Considered at the Start of the Process (Minimal Dataset)

The lists of outcomes from existing sources from both localities were reviewed and presented in our surveys using the same text/format as the original source. Unless they were described using identical terms (e.g., more than one source including ‘childhood obesity’), all outcomes were included even if they appeared to be measuring similar constructs (e.g., ‘Speech/language/communication’ and ‘vocabulary’). This resulted in a minimal dataset of N = 168 outcomes (Figure 2; Supplementary Table S1). The outcomes were subsequently grouped into eight draft COS domains by the immediate study team (Connectedness; Crime and safety; Development and education; Health behaviors; Mental health; Physical environment; Physical health; and Poverty, Social mobility and inequalities (Supplementary Table S1).

### 3.3. Delphi Studies

Following Round 1, 28 out of the 168 outcomes met the 80% threshold for automatic inclusion and were automatically included in Round 2 of the survey. According to our prespecified criteria, no outcomes could be automatically excluded following Round 1 as none had more than 80% of participants who scored 3 points or lower (=considered to be of limited importance). Overall, we noted that all outcomes received relatively high scores and were considered important by our experts (range in mean scores 5.4–8.2). This meant that to reduce the number of outcomes, while also ensuring that there were enough outcomes left across the different domains, we had to adapt our approach to include outcomes that did not meet the automatic inclusion threshold. To achieve this, we decided to include any outcome that achieved higher than 70% agreement (=% of experts giving a score of 7 or higher), rather than 80% agreement, in the second round following discussions within the research team. Additionally, we refined our list, including removing three outcomes representing the same construct as other outcomes provided responses were not dissimilar (e.g., self-confidence, removed due to presence of self-efficacy). One outcome was moved from the Physical environment domain to Development and education (language acquisition), and one outcome label was changed (from maternal physical activity to parental physical activity). These changes were made based on the expert feedback received in Round 1. Finally, one outcome domain name (‘Connectedness’) was changed to ‘Social environment’ and included outcomes from the Connectedness category, as well as four outcomes previously included under Physical environment (Figure 2).

Round 2 of the survey included 74 outcomes across 8 outcome domains. 36 outcomes were scored 7 or higher by >80% of the Delphi survey respondents and were automatically included in the community consultation. As in Round 1, no outcomes achieved the threshold for automatic exclusion. There was a discussion within the research team to decide which of the remaining outcomes should be taken forward to the next stage of the consensus process. As in Round 1, it was agreed that outcomes for which there was some consensus, but which did not reach the automatic inclusion threshold, would be included (=agreement > 70%). In addition, we chose to add back in any outcome where three or more stakeholders had suggested re-introducing an outcome that had been deleted following Round 1.

In total, 64 outcomes were taken forward for review within the community consultation. After summing up the community votes for each outcome, we found that several outcomes that ranked highly had the same number of votes. Thus, rather than having our intended ‘top 10 community-ranked outcomes’, we had 11 in Bradford and 14 in Tower Hamlets. Despite the overall similarity between the sites, some highly ranked outcomes in Tower Hamlets were considered of less importance in Bradford, and vice versa. For instance, participants in Tower Hamlets saw housing, traffic and air quality as key issues, whereas in Bradford, mental health outcomes and access to high-quality health services were brought up by many.

A comparison between the outcomes rated highly by the community and the expert agreement scores revealed that four of the most highly rated outcomes from the community consultations had not achieved 80% agreement from the experts. As planned, these outcomes were included in the final COS-EY (educational attainment, traffic, traffic levels outside schools and child weight). The remaining outcomes that were included in the community top rankings were consistent with those ranked by the experts (all achieved over 80% expert agreement) and therefore met the criteria for automatic inclusion. A total of 24 remaining outcomes that were ranked less frequently by members of the public, and where expert agreement was <80%, were removed.

### 3.4. Final COS-EY

To formulate the final COS, we once again reviewed the outcome labels and domains for clarity, including considerations of outcome hierarchy, as recommended by some of our stakeholders. An example of this is the outcome called ‘traffic’, which until this point was separate from another outcome called “traffic levels outside schools”. In the final COS-EY, these two are captured by the higher-level outcome label ‘traffic’. Overall, this process resulted in five outcomes being combined with an existing outcome, and one outcome being split into two outcomes. We reduced the number of domains from eight to six, to ensure each domain had a balanced number of outcomes (Table 2). The final COS-EY consisted of 40 outcomes, divided into six domains: Development & education; Physical health & health behaviors; Mental health; Social environment; Physical environment; Poverty & inequality (Table 2).

## 4. Discussion

This study has resulted in the development of a public health COS with six domains which can be used collectively or individually to support the evaluation of system-wide programmes designed to promote health and well-being at a population level. The COS-EY provides a set of outcomes that we recommend other evaluators adapt to align with their stakeholder priorities. We developed the COS using the ActEarly consortium as an exemplar and to support the ActEarly evaluation. There were no published COS available that were suited to our purpose, and overall, there are relatively few COS specifically designed to be used in public health interventions, particularly those delivered across a whole city [5]. There was high stakeholder agreement on the final 40 ActEarly core outcomes and the final decision on which outcomes to include was based on a large community consultation. We recommend that going forward, the COS-EY is considered for adaptation for evaluation research in this area. For ActEarly, the next step is to identify existing data sources and to decide on precise measures to assess each outcome. This work will utilize routine data collected across both study sites and aligns with the ongoing efforts to link different routine data sources [24].

### 4.1. Comparisons with Existing Outcomes Frameworks and Literature

There is a significant overlap between the COS-EY and the PHOF, which may relate to at least some of the stakeholders being aware of the existing framework; therefore, they may have used it as a point of reference when thinking about core outcomes for public health. It is important to note that, whereas the PHOF is a tool to highlight key indicators to consider, the COS-EY is a minimum set of outcomes to include in the evaluation of system-level interventions in early years and childhood settings. The overlap between the COS-EY and the PHOF means that there are publicly available data for many outcomes, including, for example, parental and child obesity, physical activity, child development, air pollution, (self-reported), well-being and homelessness. Similarly, outcomes that are included in the key indicator frameworks used by the two ActEarly local governments, (the Bradford Metropolitan District Council and the Borough of Tower Hamlets), achieved high expert consensus and are included in the COS-EY. Examples include housing, poverty and employment. Taken together, the six domains that our outcomes are categorized under (Development & education; Physical health & health behaviors; Mental health; Social environment; Physical environment; Poverty & inequality) highlight the system-wide factors that underpin early years health and well-being. The inclusion of outcomes such as ‘access to opportunity’ and ‘children get best start in life’ can be considered unique in that as far as we are aware, the existing frameworks do not include them, but both were considered highly important in our consensus work. One of the partners of ActEarly, the Bromley by Bow Centre in Tower Hamlets has further investigated the meaning of the ‘children get best start in life’ outcome and found that key elements contributing to this outcome for the Tower Hamlets community were: how families inhabit the environment and space around them; the role of play and activities for children; the stability and security needed for a firm family foundation; and the connection and support within families’ wider networks [25]. The final point raised by the communities, “connection and support within a family’s wider network”, can be understood as a systems-level outcome in that no singular measure can be expected to capture it.

There were a few unexpected exclusions that resulted from the consensus process. Breastfeeding, a key indicator in early years health research, and one of the outcomes in the PHOF that is relevant to ActEarly, was not included in the final COS-EY. Similarly, healthy life expectancy at birth, infant mortality and adverse childhood experiences (ACE), were removed. Life expectancy and infant mortality are globally tracked and are reported summary indicators that are thought to capture the overall quality of the early life period [26,27,28]. These outcomes were removed following the community consultation after failing to reach either a stakeholder consensus that was high enough for automatic inclusion, or a high priority ranking from the community. ACE were also not included in the final COS-EY despite the growing body of evidence that ACE scores are a risk factor for later-life physical and mental health outcomes, and as such, could be thought a key outcome to include in any early life research [29,30]. It is not known to us why the listed outcomes did not achieve the consensus threshold, but it could be that stakeholders felt that the interventions included in ActEarly are unlikely to result in changes in these markers of early life circumstance, or stakeholders were not familiar with the ACE concept. For community members, we think these outcomes may have felt intangible or far removed from their everyday experience—unlike other outcomes that were highly ranked (e.g., traffic). The interactions in the dot voting process are short, which means that there was not time for extensive discussions about each outcome. It is worth highlighting that our outcome sets are the minimum outcomes advocated in this area of research; thus, this does not preclude others from adding in outcomes that are deemed of high relevance even if they are not within the COS-EY.

The development of existing local government or public health outcome frameworks should include interaction with the public, rather than solely consultation with professional bodies, though this is not always done. In our community consultations, we found there was a great interest in providing researchers with feedback on what measures were meaningful to the community. The consultations further highlighted how preconceived notions held by the researchers (e.g., regarding what the most pressing public health issues are) may not reflect the lived experiences of community members. In Bradford, this became evident in the high priority given to wider, structural outcomes such as happiness and mental health, access to high-quality healthcare, employment and poverty, compared to outcomes related to diet, exercise and obesity prevention, which are some of the most pressing national and international public health priorities [31,32]. In addition, safety at home and domestic abuse were of importance for members of the public at one of our study sites and were raised despite the stigma that is commonly associated with discussing these issues [33]. It could be that the anonymity provided by our consultation method may have helped community members feel confident to give their votes to these outcomes, compared to, for example, focus groups or interview methods where the researcher knows the identity of the participant [34].

In Tower Hamlets, an important focus of discussions and responses was around housing and the issue of overcrowding (particularly during lockdown) was mentioned. Another key issue was around traffic, in particular parking and the tensions arising from it. The differences we observed between the two sites suggest that it may be advisable that researchers wishing to use and adapt the COS-EY for their own purposes start with the list of outcomes provided here, followed by some consultation with key local stakeholders to ensure they are fit for purpose.

### 4.2. Limitations

This study did not aim to achieve consensus on what the best measures or data sources are for each outcome and work needs to be undertaken before the COS-EY can be used in practice. Furthermore, some outcomes are relatively ambiguous and could be understood to mean multiple things, e.g., “access to opportunity”. There is also some overlap between different outcomes—it could be argued that diet and physical activity are very closely related to obesity and therefore not all three need to be included separately. On the other hand, obesity is a complex and multifaceted issue which does not only relate to food and physical activity (which in themselves, contribute to many things beyond an individual’s weight status), therefore, we chose not to combine these outcomes.

The Delphi process is dependent on the expert knowledge of stakeholders that are consulted [35]. This means that it cannot be considered an objective ‘truth’. With our chosen sampling strategy, it was not possible to estimate a response rate for the surveys, and therefore, we do not know who chose not to take part in the consultation and why. This was mitigated by us contacting and identifying researchers and local authority staff who were already involved with ActEarly and have a stake in selecting appropriate and meaningful outcomes. A limitation of the dot voting process was that community members only spent a few moments reading and reviewing the potential outcomes and understanding the voting process before voting and moving on. There was less time and space for more in-depth interactions and conversations with the researcher. This means there was a trade-off between low participant burden (and ease of access to the consultation) and the depth of the information we could collect from the community. In many settings in the UK, an additional barrier to community consultations can be language barriers. We mediated this in Bradford by involving a bilingual researcher in the team that collected the community consultation data. This meant that families and individuals who did not speak fluent English could ask questions about the project and the consultation in their native language. Finally, it is worth highlighting that the exclusion of outcomes from the final COS-EY does not necessarily mean that they are not of value or should not be considered by researchers. Importantly, choosing to employ the public health COS-EY in any intervention evaluation should not be taken to mean that other outcomes should not be considered, and we recommend adapting this COS to the local context wherever appropriate. For instance, if the focus of a future project was more specific than that of ActEarly, e.g., solely around the physical environment, the researchers may wish to explore that specific subset of outcomes in more depth and consult local communities on whether some of the excluded outcomes should be brought ‘back in’.

### 4.3. Next Steps

The COS-EY outcome selection to date has not been driven by what can be measured, which means that for now, we cannot be sure that all the outcomes included in the COS can be reported in a meaningful way. Therefore, and before the COS-EY can be fully implemented, we recommend that further work is undertaken to confirm the definition of each outcome, prior to deciding on the most appropriate measures or data sources. This process may be quite challenging for outcomes that cannot be captured by a single metric, but at this stage in the work, we do not think this should mean the automatic exclusion of such outcomes from the COS. Rather, we encourage further research into the area to tackle the issue of defining measures for outcomes such as ‘children get best start in life’, as clearly, these are priorities for stakeholders and communities alike. One solution to this may be the development of short lists of outcome measures that would represent each core outcome (with e.g., varying degrees of data collection burden or depending on local data availability, such as long and short versions of a questionnaire; or household vs individual level data). These lists could then be used as a starting point by investigators. While not offering perfect standardization the way a single measure per outcome would do, the process of creating lists of appropriate measures would be a step towards better standardization of public health outcomes across studies. Another avenue for future work would be to explore the relevance of the COS-EY from a policy and practice perspective and consider to what extent this work may be useful outside the research context. For instance, the COS-EY could be used by local authorities when making decisions about routine data collection practices and availability.

## 5. Conclusions

The public health COS-EY represents an initial attempt at system-wide core outcome sets developed to evaluate interventions that promote early life health and well-being, in consultation with local communities. Our chosen approach resulted in a comprehensive list of 40 outcomes, and highlighted important differences between expert knowledge and lived experience across Bradford and Tower Hamlets. Our aim was to use the COS-EY in the evaluation of the ActEarly research program in the first instance, but the COS could be applied to other settings where there is interest in evaluating early life health and well-being from a ‘wider determinants’ of health perspective.

## Figures and Tables

**Table 1:** Participants who took part in the Delphi surveys.

Participant Group	Delphi Round 1 (N Participants)	Delphi Round 2 (N Participants)
Academic	22	31
Clinical academic	3	3
Local government	5	12
Voluntary sector	2	3
Community representative	1	1
National/regional government	0	2
Commercial sector	1	0
Other ^1^	3	4
Total	37	56

^1^ This category includes people who identified their participant group as being ‘Other’ and defined it as: regional sport’s charity, clinical commissioning group, think tank, research manager and community researcher.

**Table 2:** Final COS-EY.

Core Outcome Set	Outcome Name
COS-EY 1: Development & education	1.1 Access to education
	1.2 Speech, language & communication
	1.3 Emotional & social development
	1.4 Children get best start in life
	1.5 Educational attainment
	1.6 Access to books
COS-EY 2: Physical health & health behaviors	2.1 Child physical activity
	2.2 Child sedentary behavior
	2.3 Healthy eating
	2.4 Child weight
	2.5 Childhood obesity
	2.6 Adult obesity
COS-EY 3: Mental health	3.1 Child happiness
	3.2 Child mental health (incl. children’s stress and anxiety)
	3.3 Child mental well-being
	3.4 Parental mental health
	3.5 Parental mental well-being
COS-EY 4: Social environment	4.1 Family & social relationships
	4.2 Safety at home
	4.3 Domestic abuse
	4.4 Child social relationships & bullying
COS-EY 5: Physical environment	5.1 Use, quality, and satisfaction with open space
	5.2 Parks & green spaces (incl. access to green space)
	5.3 Access to high quality health services
	5.4 Air pollution
	5.5 Food availability
	5.6 Quality of local environment
	5.7 Traffic (incl. traffic levels outside schools, parking)
COS-EY 6: Poverty & inequality	6.1 Housing (incl. homelessness; house crowding; availability of affordable housing)
	6.2 Access to opportunity
	6.3 Basic care needs met
	6.4 Employment
	6.5 Financial stability
	6.6 Inequalities
	6.7 Poverty

## Data Availability

The data presented in this study are available on request from the corresponding authors to ensure anonymity of participants.

## Supplementary Materials

The following supporting information can be downloaded at: https://www.mdpi.com/article/10.3390/ijerph19137947/s1, Table S1: Original list of 168 potential outcomes.
