# Supplementary material for: Development of Public Health Core Outcome Sets for Systems-Wide Promotion of Early Life Health and Wellbeing
Source: Int J Environ Res Public Health. 2022 Jun 28;19(13):7947. doi: 10.3390/ijerph19137947 (PMC9266033; doi:10.3390/ijerph19137947)
Supplement: Supplementary file 1 [file ijerph-19-07947-s001.zip › ijerph-1761459-supplementary.pdf]

Supplementary Materials

Table S1. Original list of 168 potential outcomes.

Outcome domain	Outcome name
Connectedness	Awareness of organised activities
Connectedness	Care provider competent, happy and kind
Connectedness	Communities driving change
Connectedness	Community connectedness
Connectedness	Community participation
Connectedness	Connection to others
Connectedness	Support to live the life I want
Crime & safety	Child social relationships/bullying
Crime & safety	Domestic abuse
Crime & safety	Safety at home
Crime & safety	Crime and anti-social behaviour
Crime & safety	Community safety
Crime & safety	Hate crime
Crime & safety	Drug dealing
Crime & safety	Perceived threat, safety
Crime & safety	Residents' perception of safety
Crime & safety	Road deaths/serious injury
Crime & safety	Victim support rate
Crime & safety	Youth justice system rates of entry
Crime & safety	Wicked problems (problems that are very challenging to resolve)
Development & education	Access to education/training
Development & education	Educational attainment
Development & education	Population education
Development & education	University/destination
Development & education	School readiness
Development & education	Vocabulary
Development & education	Speech/language/communication
Development & education	Foundational literacy
Development & education	Parental work patterns
Development & education	Parenting
Development & education	Family time
Development & education	Family support, network
Development & education	Family structure
Development & education	Family routine/habits
Development & education	Family communication
Development & education	Emotional and social development
Development & education	Development at reception
Development & education	Children get best start in life
Development & education	Childhood independence
Development & education	Child growth and development
Development & education	Behavioural development
Development & education	Attachment, bonding
Health behaviour	Active travel
Health behaviour	Organised activities (indoor/outdoor)
Health behaviour	Hobbies, activities
Health behaviour	Child physical activity/sedentary behaviour
Health behaviour	Maternal physical activity/sedentary behaviour
Health behaviour	Leisure time
Health behaviour	Adult smoking prevalence
Health behaviour	Drug use
Health behaviour	Smoking at the time of delivery
Health behaviour	Screen time
Health behaviour	Sleep
Health behaviour	Breastfeeding

Health behaviour	Initiation of breastfeeding
Health behaviour	Duration of breastfeeding
Health behaviour	Cooking skills
Health behaviour	Diet
Health behaviour	Diet knowledge
Health behaviour	Dietary preferences
Health behaviour	Dietary patterns at home
Health behaviour	Dietary patterns at school
Health behaviour	Food behaviours
Health behaviour	Food waste
Health behaviour	Healthy eating
Health behaviour	Health knowledge
Health behaviour	Healthy lifestyle knowledge
Health behaviour	Healthy lifestyle attitudes
Mental health	Adverse childhood experiences
Mental health	Aspirations
Mental health	Child belonging
Mental health	Child happiness
Mental health	Child identity
Mental health	Child mental health
Mental health	Child mental well-being
Mental health	Child self-care
Mental health	Child self-confidence
Mental health	Child self-efficacy
Mental health	Child self-esteem
Mental health	Children's stress/anxiety
Mental health	Family and social relationship
Mental health	Improving access to psychological therapies (IAPT)
Mental health	Language acquisition
Mental health	Mortality rate in persons with mental illness
Mental health	Parental happiness
Mental health	Parental mental health
Mental health	Parental mental well-being
Mental health	Provision of NICE [1] approved care package for psychosis
Mental health	Stress during pregnancy
Mental health	Subjective well-being (hedonic and an-hedonic)
Mental health	Suicide rate
Physical environment	Access to books
Physical environment	Access to education
Physical environment	Access to green space
Physical environment	Access to countryside/nature
Physical environment	Access to high quality health services
Physical environment	Access to pets
Physical environment	Access to toys
Physical environment	Air pollution
Physical environment	Air quality/CO_2 emissions
Physical environment	Allocation of spending/resources
Physical environment	Carbon footprint
Physical environment	Allotment
Physical environment	Celebrating achievements
Physical environment	Cleanliness, aesthetics
Physical environment	Climate change
Physical environment	Climate emergency response
Physical environment	Complex systems
Physical environment	Experience of accessing services
Physical environment	Fast food outlets
Physical environment	Food advertising
Physical environment	Food availability

Physical environment	Health services collaboration/Joined up working
Physical environment	Housing
Physical environment	Noise
Physical environment	Parks and green spaces
Physical environment	Quality of local environment
Physical environment	Rates of recycling
Physical environment	Residents' satisfaction with area
Physical environment	Satisfaction of where I live
Physical environment	Satisfaction with community assets/services
Physical environment	Second-hand smoking
Physical environment	Smoke in family home
Physical environment	Traffic
Physical environment	Traffic levels outside schools
Physical environment	Use of public space (lingering)
Physical environment	Use, quality, and satisfaction of open space
Physical health	Absence from work/sick leave
Physical health	Allergies
Physical health	Child height
Physical health	Child weight
Physical health	Child self-reported health
Physical health	Genetic/inherited disease
Physical health	Hearing
Physical health	Healthy life expectancy
Physical health	Life expectancy at birth
Physical health	Infant mortality
Physical health	Low birth weight
Physical health	Preterm birth
Physical health	LCT condition (lactose intolerance)
Physical health	Childhood obesity
Physical health	Adult obesity
Physical health	Oral health
Physical health	Parental self-reported health
Poverty, social mobility & inequality	Access to opportunity
Poverty, social mobility & inequality	Affordability of clubs, organized activity
Poverty, social mobility & inequality	Availability of affordable housing
Poverty, social mobility & inequality	Basic care needs met
Poverty, social mobility & inequality	Car ownership
Poverty, social mobility & inequality	Circular economy
Poverty, social mobility & inequality	Diversity of opportunity
Poverty, social mobility & inequality	Employment
Poverty, social mobility & inequality	Employment equality
Poverty, social mobility & inequality	Financial stability
Poverty, social mobility & inequality	Fuel poverty
Poverty, social mobility & inequality	Health education aspiration
Poverty, social mobility & inequality	Homelessness
Poverty, social mobility & inequality	House crowding
Poverty, social mobility & inequality	Household income
Poverty, social mobility & inequality	Inequalities
Poverty, social mobility & inequality	Living costs
Poverty, social mobility & inequality	Living in care
Poverty, social mobility & inequality	NEET [2] (16-17 yrs.)
Poverty, social mobility & inequality	Poverty
Poverty, social mobility & inequality	Social care benefit receipt and methods
Poverty, social mobility & inequality	Social mobility
Poverty, social mobility & inequality	Stability of home
Poverty, social mobility & inequality	Travel aspirations

[1] NICE stands for National Institute for Health and Care Excellence; [2] NEET stands for Not in Education, Employment or Training.
